# Idiopathic pulmonary fibrosis-specific Bayesian network integrating extracellular vesicle proteome and clinical information

**DOI:** 10.1038/s41598-023-50905-8

**Published:** 2024-01-15

**Authors:** Mei Tomoto, Yohei Mineharu, Noriaki Sato, Yoshinori Tamada, Mari Nogami-Itoh, Masataka Kuroda, Jun Adachi, Yoshito Takeda, Kenji Mizuguchi, Atsushi Kumanogoh, Yayoi Natsume-Kitatani, Yasushi Okuno

**Affiliations:** 1 Department of Biomedical Data Intelligence, Kyoto University Graduate School of Medicine, 54 Shogoin Kawahara-Cho, Sakyo-Ku, Kyoto, 606-8507 Japan; 2 Department of Artificial Intelligence in Healthcare and Medicine, Kyoto University Graduate School of Medicine, 54 Shogoin Kawahara-Cho, Sakyo-Ku, Kyoto, 606-8507 Japan; 3 Human Genome Center, The Institute of Medical Science, The University of Tokyo, 4-6-1 Shirokane-Dai, Minato-Ku, Tokyo, 108-8639 Japan; 4 Innovation Center for Health Promotion, Hirosaki University, 5 Zaifu-Cho Hirosaki City, Aomori, 036-8562 Japan; 5 Artificial Intelligence Center for Health and Biomedical Research, National Institutes of Biomedical Innovation, Health and Nutrition, 3-17, Senrioka-Shinmachi, Settsu City, Osaka, 566-0002 Japan; 6 Discovery Technology Laboratories, Mitsubishi Tanabe Pharma Corporation, 1000, Kamoshida-cho, Aoba-ku, Yokohama, Kanagawa 227-0033 Japan; 7 Laboratory of Proteomics for Drug Discovery, Center for Drug Design Research, National Institutes of Biomedical Innovation, Health and Nutrition, 7-6-8 Saito-Asagi, Ibaraki, Osaka 567-0085 Japan; 8 Department of Respiratory Medicine and Clinical Immunology, Graduate School of Medicine, Osaka University, 2-2 Yamada-Oka, Suita City, Osaka 565-0871 Japan; 9 Institute for Protein Research, Osaka University, 3-2 Yamada-Oka, Suita City, Osaka 565-0871 Japan; 10 Institute of Advanced Medical Sciences, Tokushima University, 3-18-15, Kuramoto-Cho, Tokushima City, Tokushima 770-8503 Japan; 11 Biomedical Computational Intelligence Unit, HPC- and AI-Driven Drug Development Platform Division, RIKEN Center for Computational Science, 7-1-26, Minatojima-Minami-Machi, Chuo-Ku, Kobe, Hyogo 650-0047 Japan

**Keywords:** Proteomics, Diagnostic markers, Respiratory tract diseases, Machine learning

## Abstract

Idiopathic pulmonary fibrosis (IPF) is a progressive disease characterized by severe lung fibrosis and a poor prognosis. Although the biomolecules related to IPF have been extensively studied, molecular mechanisms of the pathogenesis and their association with serum biomarkers and clinical findings have not been fully elucidated. We constructed a Bayesian network using multimodal data consisting of a proteome dataset from serum extracellular vesicles, laboratory examinations, and clinical findings from 206 patients with IPF and 36 controls. Differential protein expression analysis was also performed by edgeR and incorporated into the constructed network. We have successfully visualized the relationship between biomolecules and clinical findings with this approach. The IPF-specific network included modules associated with TGF-β signaling (TGFB1 and LRC32), fibrosis-related (A2MG and PZP), myofibroblast and inflammation (LRP1 and ITIH4), complement-related (SAA1 and SAA2), as well as serum markers, and clinical symptoms (KL-6, SP-D and fine crackles). Notably, it identified SAA2 associated with lymphocyte counts and PSPB connected with the serum markers KL-6 and SP-D, along with fine crackles as clinical manifestations. These results contribute to the elucidation of the pathogenesis of IPF and potential therapeutic targets.

## Introduction

Idiopathic pulmonary fibrosis (IPF) is a chronic, progressive interstitial lung disease categorized under idiopathic interstitial pneumonias (IIPs). IIPs represent a group of interstitial pneumonias without discernible causes and are recognized as intractable conditions in Japan. The incidence and prevalence of IPF were estimated to be 2.23 and 10.0 per 100,000 population. The incidence in men was 2.7-fold higher than that in women in Japan, which was higher than that reported by studies in the US and the UK (1.5-fold). IPF is characterized by a poor prognosis, with a median survival of 3–5 years following diagnosis and an even shorter survival, often less than 2 months, after acute exacerbation^1^. Notably, comorbidities such as ischemic heart disease, heart failure, bronchogenic carcinoma, infection, and pulmonary embolism are also significant contributors to mortality. It's crucial to emphasize that IPF patients typically exhibit poor responses to steroid treatments, and as of now, there exists no established fundamental therapeutic approach. The available pharmacological options are limited to two antifibrotic agents: pirfenidone and nintedanib. Consequently, unraveling the intricate mechanisms underlying IPF and identifying potential drug targets hold the promise of enabling more effective treatments.

In recent years, omics analysis has been actively pursued to elucidate the pathomechanisms and identify potential drug targets for IPF. Norman et al. reported an upregulation of complement iC3b in patients with advanced IPF by comparing proteomic profiles in the serum and bronchoalveolar lavage-fluid of individuals with and those without advanced IPF^2^. Additionally, Zheng et al. identified novel IPF-related biomarkers, Butyrophilin-Like Protein 9 (BTNL9) and Plasmolipin (PLLP), through an integrated analysis of transcriptomic and proteomic data from IPF patients^3^.

Three significant challenges are encountered in the pursuit of understanding disease states through omics analysis, particularly in proteomic investigations. The initial challenge pertains to the use of serum or plasma for proteome analysis, where vital biomarkers existing in trace amounts may go unnoticed due to the extensive dynamic range of proteins. To address this concern, we conducted proteome analysis of serum extracellular vesicles (EVs). Utilizing EVs enhances the capability to detect low-abundance proteins linked to pathological conditions in the bloodstream. Moreover, a noteworthy advantage of working with EVs is their capacity to encapsulate molecules that reflect pathological and disease-related status^4,5^. The second challenge lies in the fact that numerous existing omics studies, including those focusing on IPF, have predominantly concentrated on the quantifiable alterations of individual molecules^6–8^. However, to enhance interpretability and to identify more promising biomarkers and disease-associated molecules, it is imperative to explore the relationships between multiple biomolecules^9,10^. In response to this challenge, we conducted Bayesian network (BN) analyses, capable of modeling intricate combinations of multiple factors as comprehensive systems^11–14^. BN serves as a valuable methodology for examining causal relationships among variables, measuring the strength of causality through conditional probabilities, which quantify the likelihood of other events occurring when a specific event has transpired. This graphical approach is systematically employed to depict the causal connections among a multitude of events. The third challenge involves establishing connections between clinical symptoms and the underlying molecular interactions, thus enabling the investigation of disease mechanisms. Multimodal analysis, which models pathological conditions based on a variety of factors in different formats, including data from blood tests and text-based electronic medical records, in addition to omics data, has gained increasing importance^15^. Network analysis using multimodal data has been previously applied in the study of other diseases, where the integration of gene expression, genetic variation, metabolomic data, and clinical information facilitated the modeling of relationships among various factors, including critical metabolic pathways common to patients with coronary artery disease^12^. Nevertheless, these prior multimodal network analyses have failed to unveil systematic distinctions between patients and controls. Furthermore, there is no prior instance of a multimodal network analysis being conducted in the context of IPF.

BN analysis is a multivariate analytical approach employing network models to depict causal and control relationships among variables. It has demonstrated effectiveness in extracting intelligible subnetworks and discerning patient characteristics from vast networks, using Edge Contribution values (ECv) which gauge the significance of each edge for each sample, among other techniques^11,16^. In this study, we have advanced the BN and ECv methods to accommodate multimodal data and conducted a network analysis of IPF patients. The first notable aspect of this study involves the construction of a multimodal network relevant to IPF, utilizing data from proteomics, blood tests, and electronic medical records. The second aspect pertains to the quantification of edge contributions within the network for each sample, followed by network comparisons between IPF patients and control subjects. The derived subnetworks have collectively given rise to multiple modules, each associated with distinct biological functions. These modules exhibit connections to processes involving fibrosis and inflammation, effectively capturing clinical observations and serological markers characteristic of IPF. Notably, within these modules, there are candidate molecules whose associations with IPF were previously unknown.

## Results

### Study subjects and analytical strategy

**Figure 1:**
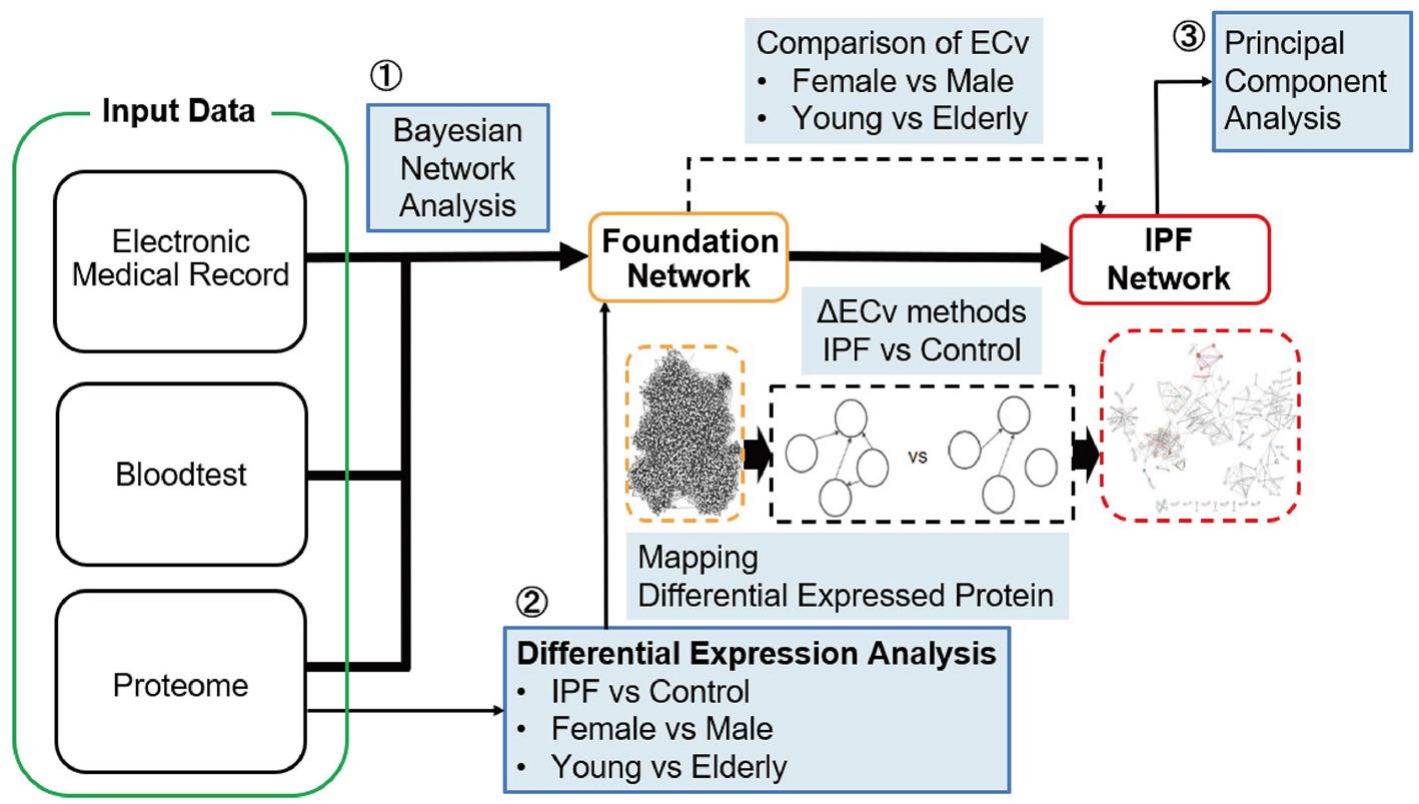
Schematic overview for IPF network analysis. We analyzed multimodal data consisting of proteome data, laboratory data and clinical characteristics by (1) Bayesian network analysis. In addition, (2) differential protein expression analysis was performed by edgeR and incorporated into the network analysis. Difference between usual interstitial pneumonia (UIP) and probable UIP (pro-UIP) was analyzed by (3) principal component analysis (PCA).

The samples, denoted as UIP and pro-UIP, were collectively analyzed as IPF. The fundamental characteristics of the study subjects are summarized in Table 1. A total of 206 patients and 36 controls participated in this study. It is essential to note that there was a significant imbalance in the dataset regarding both gender and age. The overarching objective of this research was to identify molecular networks associated with IPF, encompassing molecules with potential implications as drug targets or biomarker candidates. To achieve this objective, three distinct analyses were executed, as depicted in Fig. 1: a Bayesian Network (BN) analysis of multimodal data, which included proteomic, blood test, and electronic medical record data, followed by a differential expression analysis (DEA) of the proteomic data. The findings from the DEA were subsequently integrated with the results from the BN analysis, and Principal Component Analysis (PCA) was applied to both the ΔECv values calculated by the BN analysis and the DEA data. The term "ΔECv" denotes the absolute difference in ECv for each edge when comparing samples under distinct conditions.

**Table 1:** Characteristics of the study subjects.

	Control	IPF patients	*P* value
Number	36	206	
Age, mean (SD)	67.28 (12.68)	72.86 (9.33)	0.002
Male, n (%)	17 (47.2)	154 (74.8)	0.002

### IPF-specific proteome networks identified by BN analysis

The foundational network was derived from the combined dataset of all samples, encompassing both patients and controls. The ultimate structure of this foundational network comprised 2594 nodes and 14,861 edges, demonstrating an average node order of 11.46. A detailed breakdown of the edges that constitute the estimated foundational network is provided in Supplemental Table 1. We selected edges representing the top 1% in terms of the magnitude of ΔECv for each type of child node, taking into account the number of edges within the foundational network.

**Figure 2:**
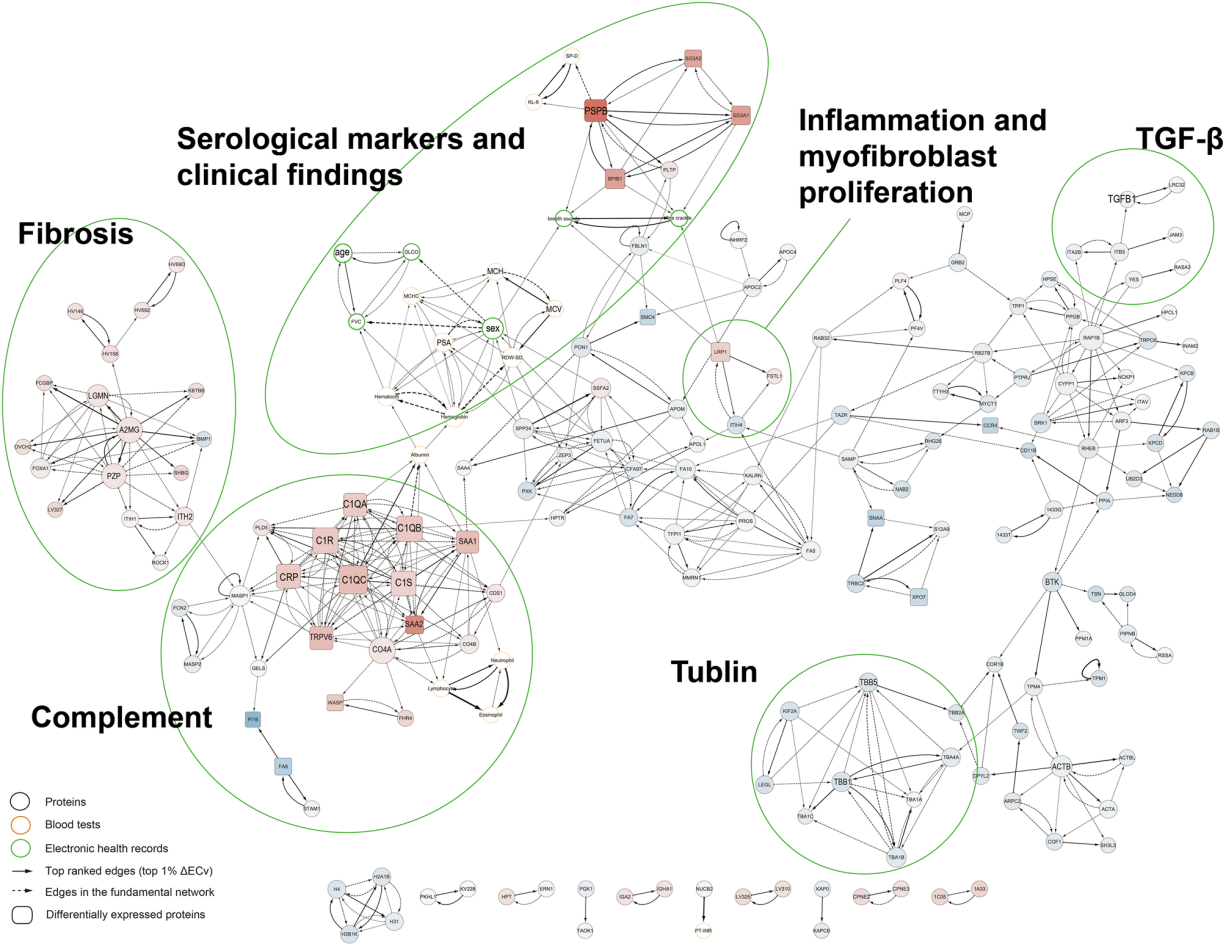
IPF network extracted by the ΔECv method. Details are described in Fig. 3.

Consequently, we extracted 131 edges whose children were protein nodes, 13 edges associated with blood test nodes, and 4 edges related to electronic medical record information. These 148 selected edges were further mapped and interconnected within the foundational network, culminating in the creation of a subnetwork that characterizes IPF, which we refer to as the "IPF network." This IPF network comprises 184 nodes and a total of 511 edges, as illustrated in Fig. 2.

The IPF network is organized into various modules, each composed of functionally related proteins. Alongside clinical findings and serum markers that are characteristic of IPF, this network incorporates newly identified candidate proteins. However, it remains unclear how the results from Bayesian network analysis (BNA) are associated with the individual expression of proteins.

**Figure 3:**
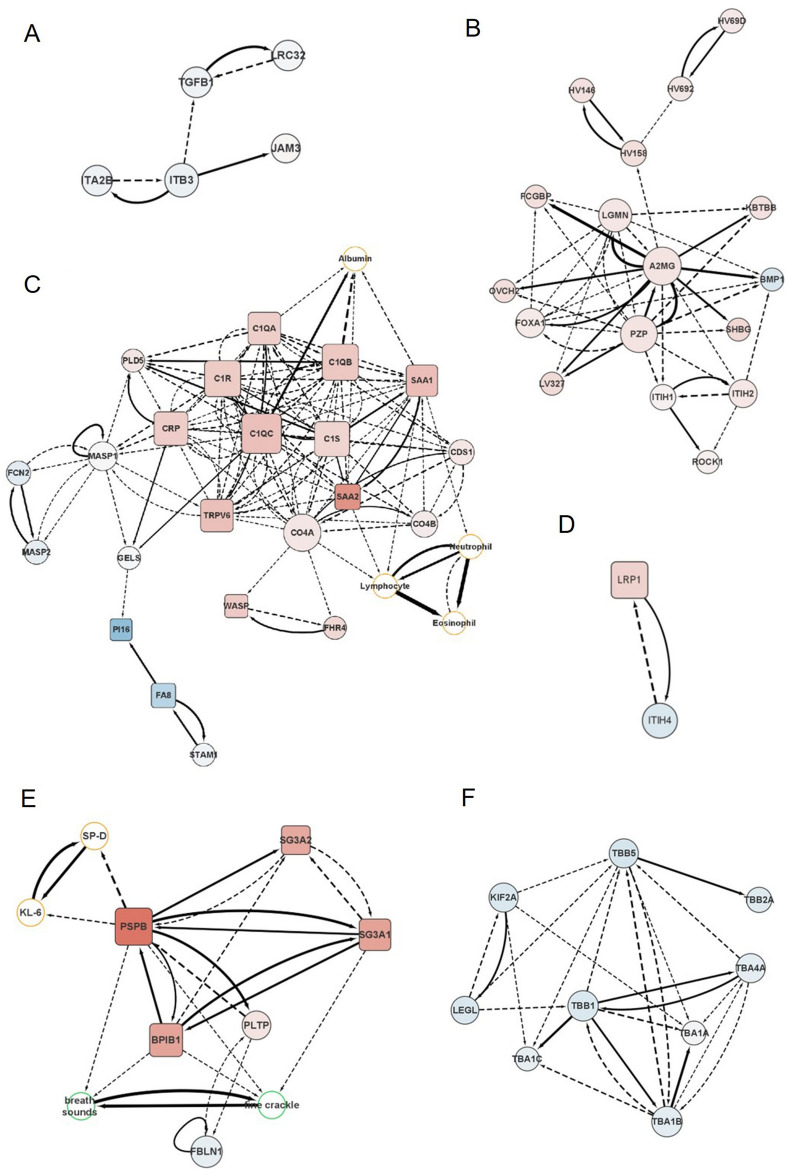
Enlarged views of modules in the IPF network. Key components of IPF-specific network were extracted as modules including (**A**) TGF-β-related module, (**B**) Fibrosis-related module, (**C**) Complement-related module closely connected with fibrosis-related module, (**D**) Module related to inflammation and myofibroblast proliferation, (**E**) Module related to serological markers and clinical findings, and (**F**) Tubulin-related module. Several clinically relevant biomarkers were identified such as PSPB (**E**), a surfactant protein essential for lung function, which was connected with serum levels of SP-D, KL-6 and clinical manifestation of fine crackle. Solid lines are edges selected under the condition of the top 1% of ΔECv, dotted lines are edges connected to selected nodes and edges at distance = 1. The width of the edge reflects the size of ΔECv. Square nodes represent differential expressed proteins between the IPF group and control group. The size of nodes represents the number of edges leaving from the node, namely the number of outgoing orders. Black nodes represent protein, orange nodes represent blood tests, green nodes represent EHR. Nodes painted red inside represent proteins upregulated in the IPF group compared to the control group, blue inside represents downregulated.

Bayesian network depicted clinically relevant IPF-specific networks, which are mainly consisted of 6 modules including TGF-β-related module (major components: TGF-β and LRC32 proteins), fibrosis-related module (A2MG and PZP proteins), complement-related module (C1QC and SAA2 proteins; neutrophil, eosinophil and lymphocyte counts), module related to inflammation and myofibroblast proliferation (LRP1 and ITIH4 proteins), module related to serological markers and clinical findings (PSPB protein; serological markers, KL-6 and SP-D; and fine crackles), and tubulin-related module (TBB1 and TBB5). Details of the modules are shown in Fig. 3. The complement-related module is connected with the fibrosis-related module by MASP1 and ITIH2, and it is linked with the module related to serological markers and clinical findings via serum albumin. Age and sex are not linked with proteins, rather they are linked with a hemoglobin level or respiratory function (flow-controlled ventilation, FCV). Solid lines are edges selected under the condition of the top 1% of ΔECv, dotted lines are edges connected to selected nodes and edges at distance = 1. The width of the edge reflects the size of ΔECv. Square nodes represent differential expressed proteins between the IPF group and control group. The size of nodes represents the number of edges leaving from the node, namely the number of outgoing orders. Black nodes represent protein, orange nodes represent blood tests, green nodes represent EHR. Nodes painted red inside represent proteins upregulated in the IPF group compared to the control group, blue inside represents downregulated.

### Integrated view of differential expressed proteins into the IPF network

Differential expression analysis (DEA) was conducted on the 2410 proteins employed for network estimation. Among these, a total of 123 proteins were identified with an FDR < 0.05 and |logFC| > 1.0. Out of these, 100 proteins exhibited upregulation in IPF patients, while 23 were downregulated. The results of molecular function analysis using IPA revealed significant enrichment of molecular functions related to post-translational protein modification, cellular assembly, organization, and cellular compromise within these differentially expressed proteins (Supplemental Fig. 1). Furthermore, these differentially expressed proteins were integrated into the IPF network. An overview of the final IPF network is shown in Fig. 2.

### Identification of novel protein networks

The IPF network comprised several functional modules, encompassing factors pertinent to TGF-β signaling, fibrosis, myofibroblasts and inflammation, complement activation, serum markers, clinical manifestations, and tubulin (Fig. 2). TGF-β, a well-known factor elevated in IPF patients, demonstrated significant differences in ECv, particularly between TGF-β and LRC32 in cases versus controls (Fig. 3A). At the core of the fibrosis-related module, A2MG (alpha-2-Macroglobulin) and PZP (pregnancy zone protein) were notably positioned (Fig. 3B). The complement-related module (see Fig. 3C) was in close proximity to the fibrosis-related module, and their connection was mediated by MASP1 (Mannan-binding lectin serine protease 1). Modules related to myofibroblast and inflammation featured LRP1 (Prolow-density lipoprotein receptor-related protein 1) and ITIH4 (Serum Inter-Alpha-Trypsin Inhibitor Heavy Chain 4) (Fig. 3D). Additionally, modules associated with serum markers and clinical manifestations included KL-6 (Sialylated carbohydrate antigen KL-6), SP-D (Pulmonary surfactant-associated protein D), and the presence of fine crackles (Fig. 3E). Tubulin-related modules featured various tubulin components (Fig. 3F). For a more detailed view of each module within the IPF network, please refer to Fig. 3. Furthermore, molecular function analysis was conducted using protein expression data from the IPF network to explore the biological relevance of the extracted subnetwork (Supplemental Fig. 1). Significantly, "Cellular Movement" and "Cellular Assembly and Organization" emerged as notable molecular functions represented by proteins in the IPF network. Additionally, these proteins in the IPF network were associated with infectious diseases and conditions related to inflammatory responses.

### Comparison of networks of UIP, pro-UIP, and control

The diagnosis of UIP and pro-UIP was based on HRCT imaging and histopathology in accordance with the official ATS/ERS/JRS/ALAT Clinical Practice Guideline of IPF Diagnosis and Treatments^1^, Consequently, both UIP and pro-UIP are confidently considered as IPF. However, acknowledging the potential for pro-UIP to encompass diagnoses beyond IPF, we also conducted an analysis that excluded pro-UIP from the IPF category. Notably, the UIP-specific network (Supplemental Fig. 2) exhibited the same modules that were identified in the IPF-specific network. However, it is worth mentioning that the connections among these modules were more prominently illustrated in the IPF-specific network.

**Figure 4:**
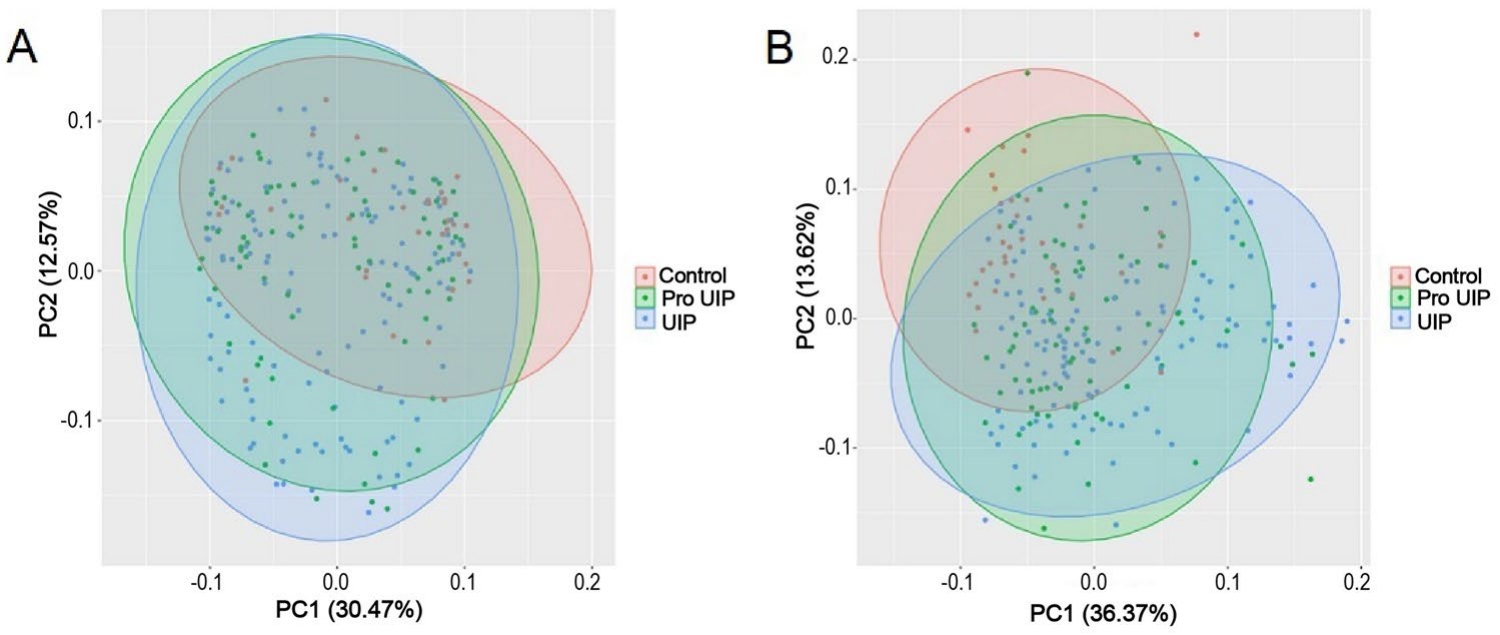
PCA based on the ECv matrix and the expression values. (**A**) PCA of the IPF and control groups using ECv. One hundred and thirty-one Edges connecting protein to protein were selected from the edges narrowed down under the ΔECv top 1% in the IPF vs. control groups. A standardized ECv matrix was used in the analysis. Each colored oval represents a 95% confidence interval. UIP and control showed significant differences (p = 0.002). Distribution of pro-UIP was close to that of UIP, but slightly close to the distribution of control. (**B**) PCA of the IPF and control groups with expression values of differential expressed proteins. From the protein nodes that comprise the IPF network, the 21 proteins that satisfy FDR < 0.05 and |logFC| > 1.0 in the comparison of the IPF vs. control groups were standardized and used in the analysis. Each colored oval represents a 95% confidence interval. UIP and control only showed significant differences (p = 0.0001). *PCA* principal component analysis.

We further attempted to identify unknown pathological subtypes using PCA, employing protein expression and the ECv matrix as inputs, as illustrated in Fig. 4. In the case of PCA based on protein expression values, we focused on proteins that exhibited significant differences in the differential expression analysis and selected the 21 proteins contained within the IPF network. For PCA based on the ECv matrix, we applied constraints to the edges between proteins to mitigate noise, ultimately utilizing the 131 edges found within the IPF network.

In the plot generated from the first and second principal components, samples labeled as UIP/pro-UIP/control exhibited distinct distributions in both protein expression and the ECv matrix. Consequently, we conducted an analysis to determine whether there were statistically significant differences in the distribution of each group using PERMANOVA (Permutational multivariate analysis of variance). The input is a euclidean distance matrix based on the expression values, and the number of permutations is set to 1000.

A comparison of the three groups, namely UIP, pro-UIP, and control, revealed significant differences in distributions between the UIP and control groups (protein expression: p = 0.0001, R^2^ = 0.02276, ECv matrix: p = 0.002, R^2^ = 0.01652). However, it's important to note that distinguishing IPF from the control group based solely on these results is challenging due to partial overlap in the distributions of these three groups. Nonetheless, this outcome does underscore the distinct characteristics of each group in terms of both individual protein expression and the biological system. It suggests that the ECv variation captures group-specific features to a certain extent. Particularly noteworthy is the positioning of the pro-UIP group between the UIP and control groups, which may reflect the unique characteristics of each group. Furthermore, we identified a patient subgroup with distributions that do not overlap, implying the potential existence of an unknown subtype.

### Stratified analysis by sex and age

As shown in Table 1, the dataset exhibited significant disparities in the male-to-female ratio and in the distribution of younger and older individuals between IPF patients and controls (p < 0.05). It has been previously documented that pulmonary function in IPF patients may vary according to gender^17,18^, and age is a significant factor affecting the development of IPF and subsequent survival duration of the patients^19^.

Therefore, we conducted a stratified analysis and visualized the differences in protein expression and network using stratified analysis by sex and age.

Using the Mann–Whitney U test and Bonferroni correction, we identified 5 edges (1.0% of 511 edges in the IPF network) specific to male IPF patients, with 6 edges (1.2%) common to both male and female patients (Supplemental Fig. 3A). In the IPF network, we observed certain modules, particularly centered around PSPB (Pulmonary surfactant-associated protein B), which displayed notable variability based on sex. However, the majority of the IPF network exhibited little variation between the sexes.

Next, we examined network differences based on age, categorizing individuals under 65 as "young" and those over 65 as "elderly." This analysis revealed that 11 (30.6%) IPF patients and 28 (13.6%) control subjects fell into the "young" category. Similar to the findings regarding sex differences, a stratified analysis was conducted. We identified 13 edges (2.5% of 511 edges in the IPF network) that are characteristic of elderly IPF patients, with 6 edges (1.6%) common to both elderly and young patients. These results were found to have a corrected p-value of less than 0.05 (refer to Supplemental Fig. 3B). An intriguing observation is that the edges showing differences between elderly and young individuals closely mirrored those that differed between males and females. This suggests that the majority of the extracted IPF network represents a common underlying mechanism that is not significantly influenced by sex or age.

## Discussion

Our study has demonstrated that circulating EVs serve as a valuable source of biomarkers for diagnosing IPF. Through BN analysis, we successfully identified both novel and established biomarkers, including well-known molecules such as TGF-β. Employing a multimodal approach that integrates proteomics, blood tests, and electronic medical records, we gained a deeper understanding of the biological and clinical significance of these proteomic markers. The IPF-specific network comprises several modules, including those related to "fibrosis," "inflammation, and myofibroblast proliferation," "complement", "serological markers, and clinical findings," and "tubulin." The biological interpretation of each module has provided valuable insights into the pathogenesis of IPF.

The IPF network includes TGF-β1, a well-known factor that is upregulated in IPF patients. TGF-β1 plays a crucial role in mobilizing fibroblasts to the site of tissue injury, driving their differentiation into myofibroblasts, and subsequently stimulating the production of extracellular matrix (ECM) by these myofibroblasts^9^. TGF-β, along with various ECM proteins, promotes fibrosis^19^. LRC32 (GARP), directly linked to TGF-β1 in the BN, is recognized for binding to TGF-β and controlling its distribution and signal transduction^20^. We also identified a fibrosis-related module that is distinct from the TGF-β module. A2MG and PZP are two molecules with structurally similar features, both capable of binding to TGF-β. While A2MG has frequently been associated with liver fibrosis and considered a strong candidate biomarker^21^, its connection to lung fibrosis^22^, and potentially IPF mechanisms, has been less reported. Importantly, A2MG and PZP, though initially overlooked in differential expression analysis, emerged as pivotal molecules through the network analysis.

Another interesting module is related to complement and inflammation, which locates close to the fibrosis-related module (Fig. 3C). Upregulation of complement-associated factors is thought to promote inflammation^23^. The major acute phase proteins, SAA1 (Serum amyloid A-1 protein) and SAA2 have cytokine/chemokine-like properties and they can be expressed at sites of inflammation and recruit inflammatory cells. In accordance with this, BN showed that SAA1 and SAA2 are linked with immune cells including lymphocytes, neutrophils. These proteins are reported to be related to lung fibrosis. However, since changes in the expression of these proteins are not specific to IPF, combinations of biomarkers need to be considered^7^. Factors related to complement and inflammation, including SAA1 and SAA2, were connected by edges to each other, forming a single module. Similarly, A2MG and PZP belonged to a module consisting of the module involved in fibrosis. Fibrosis-related modules and complement-related modules were connected by MASP1. It works in the complement lectin pathway, and at the same time, it forms a complex with A2MG, a key molecule in the fibrosis-related module^24^, confirming that our network model is reliable. Actually, MASP1 and A2MG were linked by ITIH2. ITIH2 is a protein that binds to hyaluronic acid and is a component of serum Inter-Alpha-Trypsin Inhibitor (ITI) that may contribute to angiogenesis^8^.

On the other hand, we identified a module that appears to be involved in both inflammation and fibrosis (Fig. 3D). LRP1 is an endocytic receptor that works in endocytosis and phagocytosis of apoptotic cells^25,26^. Dysregulation of LRP1 expression has been suggested to cause fibrosis through the release of TGF-β from ECM and promoting the proliferation of contractile myofibroblast^27^. ITIH4, similar to ITIH2, is an acute phase protein involved in inflammatory response to injury^28^. It has been reported to be significantly correlate with the severity of liver fibrosis^29^, suggesting its potential role in IPF.

The IPF network also included well-known serological markers and clinical findings (Fig. 3E). KL-6 is a serological marker used for the diagnosis of interstitial pneumonia. SP-D is recognized as a lung-specific serological marker, associated with the respiratory function and prognosis of patients with interstitial lung disease^30,31^. Fine crackles, a valuable auscultatory finding for IPF screening^32^, were connected to PSPB in the IPF network. PSPB is a crucial protein that contributes to alveolar stabilization, and its deficiency causes acute respiratory distress syndrome^33^. Normally, PSPB is scarcely found in the serum but increases when alveolar capillaries are damaged due to lung inflammation and fibrosis^34^. In consistent with previous reports, this study also observed a significant increase in PSPB in IPF patients compared to the control group.

Modules related to complement and fibrosis were upregulated overall, while tubulin components such as TBB1 and TBB5 were downregulated. It is previously documented that β-tubulin experiences reduced expression in the lower respiratory tract of cystic fibrosis patients, associated with an increase in vimentin-positive cells, implying the occurrence of epithelial-to-mesenchymal transition (EMT)^35^. In the context of IPF, acetylation of α-tubulin is thought to be implicated in the disease's pathogenesis^36^. Regarding the interactions among these modules, the complement-related module reveals extensive interconnections with other modules, functioning as a central hub. This suggests a plausible pathogenic mechanism in which complement and SAA2 recruit inflammatory cells, initiating the onset of the disease. Subsequently, inflammation propagates, and fibrosis is induced as a secondary effect, influenced by factors such as TGFB and LRP1. Given the pivotal role of complement within this network, molecules associated with this pathway may hold promise as therapeutic targets. Consequently, the intricate interplay within the network offers significant added value.

The literature has previously reviewed potential protein biomarkers in the plasma of IPF patients^37^, broadly categorizing them into three groups: (1) complement and chemoattractant factors (C1R, CCL17, CXCL12, A100A1, ficolin-2), (2) extracellular matrix proteins (actin, cytoplasmic 2, ECM1, and fibronectin), and (3) coagulation factors (antithrombin III, kininogen 1). Our study successfully validated the significant association of C1R with IPF, which aligns with the findings that the complement-related module, inclusive of the chemoattractant SAA2, plays a central role in the IPF-specific network. Looking ahead, it may become possible to diagnose IPF from peripheral blood using Enzyme-linked Immuno-sorbent Assay (ELISA), enabling early diagnosis, disease monitoring, and assessment of treatment effectiveness. Notably, if a patient's blood analysis reveals an elevation in SP-D, this could potentially open the door to targeted therapies focused on PSPB, given the observed association between SP-D and PSPB protein.

Subnetwork extraction using ECv comparisons successfully integrated proteins associated with lung function and fibrosis, along with serological markers and clinical findings specific to IPF, into a subnetwork. Our findings illustrated that the IPF network encompassed several molecules that were not identified through differential expression analysis, highlighting the benefits of the ΔECv method, which places emphasis on disparities in causal relationships between variables.

Given that these novel molecules were interconnected with well-established biomarkers, the criteria applied in this study effectively isolated disease-specific subnetworks. Nonetheless, it's crucial to note that our study did not directly establish the causal relationship of these markers with IPF. Further investigations are warranted to validate their clinical and pathophysiological significance.

Another advantage of network analysis is its capacity to explore various facets of confounding factors, which are frequently encountered in the analysis of medical data. In our study, we identified edges that exhibited significant variability by sex and age and assessed their influence on the IPF network. By incorporating sex and age nodes within the network, we aimed to account for the potential impact of population bias on the analysis. Interestingly, age and sex were not found to be directly associated with extracellular vesicle protein levels; instead, they were linked to variables like hemoglobin levels and respiratory function. In the sex- and age-stratified analysis, it was observed that the fibrosis-related module and the inflammation and myofibroblast proliferation module were distinctive features among male and younger patients. Considering that men are more susceptible to IPF, these modules might be linked to the acceleration of the disease phenotype in these groups. It's worth noting that discussions regarding sex and age differences in IPF are currently limited. A more detailed analysis with a specific focus on these factors is warranted to achieve a deeper understanding of the disease and advance personalized medicine.

Additionally, we utilized a multimodal dataset, integrating data with distinct properties for network analysis, aiming to capture the distinctive features of IPF. There are two significant advantages in positioning elements like serological markers and clinical findings within omics networks. Firstly, it enhances the model's interpretability. For instance, we observed links between PSPB, a pulmonary surfactant protein, and BPIB1, associated with bactericidal infection, and mucosal permeability, and fine crackles. These connections provide valuable insights into the network's structure. Secondly, elements frequently measured in diagnosis and treatment monitoring can serve as surrogate markers for proteins. For instance, a complement-related module is intricately connected to the nodes representing neutrophils and lymphocytes. This network structure effectively illustrates the close molecular relationship between complement and leukocytes^38^, and serves as a reminder of the significance of monitoring leukocyte counts in patients with IPF. In prior studies, the connections between diverse modal factors, like respiratory function measurements and blood test results, were typically explored using correlation coefficients between these factors^6,7^. In contrast, BN analysis enabled us to holistically assess the interconnections among multimodal factors. Moreover, it provided us with the ability to affirm the clinical significance of the factors linked by edges within the network.

Lastly, we endeavored to uncover an unidentified subtype through PCA, utilizing protein expression and the ECv matrix, which includes the ECv values for each edge in the IPF network for each sample, as inputs. Not only did we observe significant distinctions in the distributions between the IPF patients' group and the control group, but we also identified a subgroup of IPF patients with non-overlapping distributions. This discovery implies the presence of a novel subtype. While gender and age did not have a significant impact on the network, our data indicated that the weights on individual networks vary between individuals. Bayesian network analysis allows for the depiction of networks for each individual, making it possible to select targeting molecules with significant influence. In the future, by considering the network perspective, strategies such as targeting multiple molecules or focusing on hub molecules that connect the network could be realized. This could open avenues for personalized therapies based on the distinct molecular profiles of individuals.

There are several limitations in the present study. First, there was an age and sex mismatch between patients with IPF and control subjects. However, stratification analysis revealed that sex-specific networks and age group-specific networks were primarily observed in the “serological marker and clinical findings” module. This suggests that the IPF network identified in this study is a shared characteristic among IPF patients, regardless of age and sex. Although we detected a few differentially expressed proteins specific to males or females, the majority of the edges were common to both sexes. This can be advantageous when comparing the average of ECvs in each sample. It might be necessary to extract subnetworks in a more context-sensitive manner, such as adjusting the ΔECv threshold depending on the specific module. Second, our results lack replication in an independent cohort. Third, we did not conduct functional analyses of the biomarkers identified in this study. Therefore, replication studies and functional investigations are necessary to validate the significance of the biomarkers identified in our research. In future studies, it would be desirable to prospectively recruit IPF patients and controls and quantify the target proteins identified in the present study to investigate their associations with clinical indicators. Our strategy could also involve the collection of serial blood samples over time to explore their relationship with disease severity. Additionally, including different ethnic populations in the study would be optimal. For functional analysis, a disease model using patient-derived induced pluripotent stem (iPS) cells may be useful in determining whether the changes in extracellular vesicle proteins observed in patients can be replicated. Subsequently, inhibition experiments can be employed to pinpoint the causal factors and validate the efficacy of proposed treatment strategies. One specific approach could involve generating lung organoids from iPS cells and examining their effects on fibrosis, either using a model of fibrosis induced by substances like BLM or leveraging protein data from exosomes released by these organoids. Another in vitro functional evaluation method would be to investigate whether EMT (an in vitro model of fibrosis) is enhanced by the co-expression (forced expression) of the protein in human airway epithelial cells. Lastly, it's worth noting that we used proteins derived from serum EVs, not proteins from alveolar lavage fluid. Confirming the correlation between these two sources would further support the validity of the study.

## Conclusion

In this study, we succeeded in capturing IPF-specific biomarkers that are associated with the inflammatory and fibrosis systems via multimodal BN analysis using ECv. By integrating blood test data and electronic medical record data into the network, protein biomarkers became clinically interpretable, and the annotation of the proteome networks became easier. We could visualize the relationship of many IPF-related molecules that have been reported individually in the past. Furthermore, we identified several novel biomarkers that were overlooked by differential expression analysis. Although replication studies are needed to draw conclusions, this study has shed light on a portion of the molecular network and modules related to IPF. Thus, in the future, strategies such as combination therapy for different modules and targeted treatments tailored to symptoms and disease subtypes can be considered. Further research is required to deepen the understanding of the pathogenesis of IPF and to develop new treatment strategies.

## Materials and methods

### Study subjects

All methods of this study were performed in accordance with the Declaration of Helsinki and relevant named guidelines and regulations. The study was approved by the Ethics committee of Osaka University (approval number 187). Written informed consent was acquired from all patients before this study. The protocol of this study was approved by the Ethics Committee of National Institutes of Biomedical Innovation, Health and Nutrition (Approved Number: 187) and Osaka University Hospital (Approved Number: 18315). Six hundred and two samples with pulmonary fibrosis who were treated at Osaka University Hospital participated in the study. Those who did not show any organic respiratory abnormality as a result of the examination were considered healthy. Patients were classified according to the Official ATS/ERS/JRS/ALAT Clinical Practice Guideline^1^ as "usual interstitial pneumonia (UIP) ", "probable UIP (pro-UIP)", "indeterminate for UIP", or "alternative diagnosis" according to the information of HRCT (High-Resolution Computed Tomography) image findings and histopathological and clinical findings. The dataset included a proteome measured comprehensively from serum EVs (2445 items)^39^, blood test information (161 items), electronic medical record information (53 items) and CT scan findings. After excluding those with missing data, 591 samples were used for network estimation, including 206 IPF patient samples and 36 controls.

### Acquisition of medical information

Medical information securely stored in the data center of Osaka University Hospital was anonymized by patient ID and then stored in encrypted HD with the cooperation of the Medical Information Department of the Osaka University Hospital and provided to the National Institute of Biomedical Innovation (NIBIO). Medical examination records were obtained as structured data from the doctor using a template created with a list of 102 items of necessary information in advance, or by manually curating the template from free text data at the NIBIO. The CT imaging interpretation reports were tagged with key words using manual or natural language processing techniques, and were classified into site/lesion pairs and three categories: positive, negative, and suspect. Blood test values were structured by selecting and curating 173 key items. For the initial medical questionnaire and basic information, the key items were curated and added to the template items of the medical record. In structuring the data, we confirmed the meaning of missing values and used mainly the reference values for healthy subjects to impute missing values, referring to materials from the Japan Society for Clinical Laboratory Science and the laboratory department of Osaka University Hospital.

### Sample collection, purification of extracellular vesicles

Ten mL of blood was collected and allowed to stand at room temperature for 1 h, then centrifuged at 3000 rpm for 10 min, and the supernatant was separated as serum. The separated serum was immediately frozen and stored in a freezer at -80 °C. Serum was also collected in the same manner for those who were diagnosed as having no organic respiratory disease as healthy control. EV isolation and comprehensive protein measurements were performed according to the method described in Muraoka et al.^40^. Briefly, phosphatidylserine-positive extracellular vesicles were purified from 200 μl of serum using MagCapture isolation kit (Fuji lm Wako). Proteins in EVs were reduced with tris(2-carboxyethyl) phosphine, alkylated with iodoacetamide, trypsin digested and desalted.

### Proteome analysis

As described previously^39^, pretreated samples were subjected to LC–MS/MS analysis using the Data independent acquisition (DIA) method^40^. Data analysis was performed using DIA analysis software Spectranout, and run-wise imputation was performed for missing values. One commercial serum sample was added to every 15 samples as a quality control to assure quality from sample preparation to data analysis. DIA analysis of digested HeLa cells was also performed as a quality control for mass spectrometry.

The proteome data were log-transformed (base: 10) converted to logarithms based on their expression intensity prior to visualization. The heatmap was created with seaborn python module with the parameter settings as below: method = 'average', metric = 'cosine', z_score = 1, standard_scale = None. For t-SNE and UMAP, the proteome data was further converted into z-score. The t-SNE was conducted with the scikit-learn python module with the parameter settings as below: n_components = 2, perplexity = 5, metric = ’cosine’. The UMAP was conducted with the umap python module with the parameter settings as below: n_components = 2, n_neighbors = 5, metric = ’cosine’. Among 2445 items, 35 duplicated IDs were excluded and 2410 were used for network estimation.

### Bayesian network analysis and estimation of IPF-specific network

The data were distributed differently for each test item (proteome, blood tests, and electronic medical records) and contained both continuous and discrete values. Therefore, standardization was used to solve this problem. This enables network analysis, which integrates a variety of data. Subnetwork extraction specific to IPF patients was performed following the previously described method^11,41^. Briefly, Bayesian network (BN) was utilized to represent conditional independence among variables and estimate causal relationships. Foundation network estimation, shared by both IPF patients and controls, was conducted using BN and the B-spline nonparametric regression model. The NNSR algorithm enabled BN estimation from large datasets by iteratively estimating subnetworks. The regression model's parameters were re-estimated using B-spline based on the structure obtained from the NNSR algorithm. ΔECv was calculated to compare network differences between conditions, such as IPF patients and controls, and edges with larger ΔECv values were selected for further analysis. While a threshold value of 1.0 for ΔECv was used in previous analyses, the current study's small ΔECv values led to the selection of the top 1% for network narrowing. The subnetworks with the top 1% ΔECv values are considered characteristic of IPF patients. Cutoff value for the estimated frequency was set at 0.05. The number of subnetwork estimation iterations (denoted as T) was set to T = 100,000, and network estimation was performed three times under identical conditions. Edge coincidence was calculated for each pair of estimated networks, and it was confirmed that, on average, more than 95% of the network structures were consistent. This indicates that the estimated network has a sufficiently stable structure. The final foundation network structure was obtained by removing nodes and edges errored in the computation of the parameters of the model necessary for calculation of ECv. Visualization of the estimated network was performed using Cytoscape^42^.

Network estimation specific for IPF was performed using ECv value that quantifies the importance of a particular edge for each sample. Patients with similar molecular systems have similar ECv. Furthermore, edges, where ECv differs significantly between samples under different conditions, can be considered characteristic edges between the two groups in the systems captured by BN. Therefore, by extracting edges based on the differences in ECv between different samples, we can extract subnetworks that are differentiated between samples in different conditions. Here, by comparing the ECv of the IPF patient samples and the ECv of the controls for all edges of the foundation network, we extracted edges that differ significantly in IPF between the two groups.

### Differential expression analysis for proteins

Differential expression analysis for the 2410 proteins used for network estimation was performed using library edgeR^43^ and in R 4.1.0 (R Core Team, 2018). Differential expression Proteins were defined as those meeting both FDR (False Discovery Rate) < 0. 05 and |logFC (log Fold Change)| > 1.

### Molecular function analysis

Ingenuity Pathway Analysis^44^ was used for molecular function analysis.

### Principal component analysis

Principal component analysis (PCA) was used to visualize the difference of protein expression without normalization or ECv among UIP patients, pro-UIP patients and controls. The R packages ggplot2^45^ and ggfortify^21^ were used for PCA. The R package vegan (https://github.com/vegandevs/vegan) was used for PERMANOVA (Permutational multivariate analysis of variance).

### Computational environment

INGOR. 0.14.0 (A newer version of SiGN-BN; https://ytlab.jp/clinfo/ingor/index.html was used for Bayesian network estimation^40^. The super-computing resource was provided by the Human Genome Center, the Institute of Medical Science, the University of Tokyo (http://sc.hgc.jp/shirokane.html).

### Supplementary Information

Supplementary Information.

## Data Availability

The proteome data used in this study are available in jPOST Database (https://globe.jpostdb.org/) with the accession number of PXD042707 (https://repository.jpostdb.org/preview/72738941464e7f28bc3fe4, Access key: 6478).

## Abbreviations

IPF: Idiopathic pulmonary fibrosis
IIP: Idiopathic interstitial pneumonia
BN: Bayesian network
ECv: Edge contribution value
UIP: Usual interstitial pneumonia
Pro-UIP: Probable UIP
HRCT: High-resolution computed tomography
PCA: Principal component analysis
DEA: Differential expression analysis
PERMANOVA: Permutational multivariate analysis of variance
