# Supplementary material for: Idiopathic pulmonary fibrosis-specific Bayesian network integrating extracellular vesicle proteome and clinical information
Source: Sci Rep. 2024 Jan 15;14:1315. doi: 10.1038/s41598-023-50905-8 (PMC10789725; doi:10.1038/s41598-023-50905-8)
Supplement: Supplementary file 1 — Supplementary Information. [file 41598_2023_50905_MOESM1_ESM.docx]

**Idiopathic pulmonary fibrosis-specific Bayesian network integrating extracellular vesicle proteome and clinical information**

**Supplemental materials**

**Supplemental figure 1. Molecular function analysis by Ingenuity Pathway Analysis.**

Molecular function **(A)** and diseases **(B)** which is related to IPF-specific proteins with high logFold Change (logFC) were shown. LogFC values for each proteome item included in the IPF network were used as an input. Each bar chart shows the association between dataset and category. The vertical axis represents the -log (p-value) in Fisher’s Exact Test and the orange line represents Significance＝-log (0.05). "Cellular Movement", "Cellular Assembly and Organization" like molecular functions were observed significantly more in proteins in the IPF network.


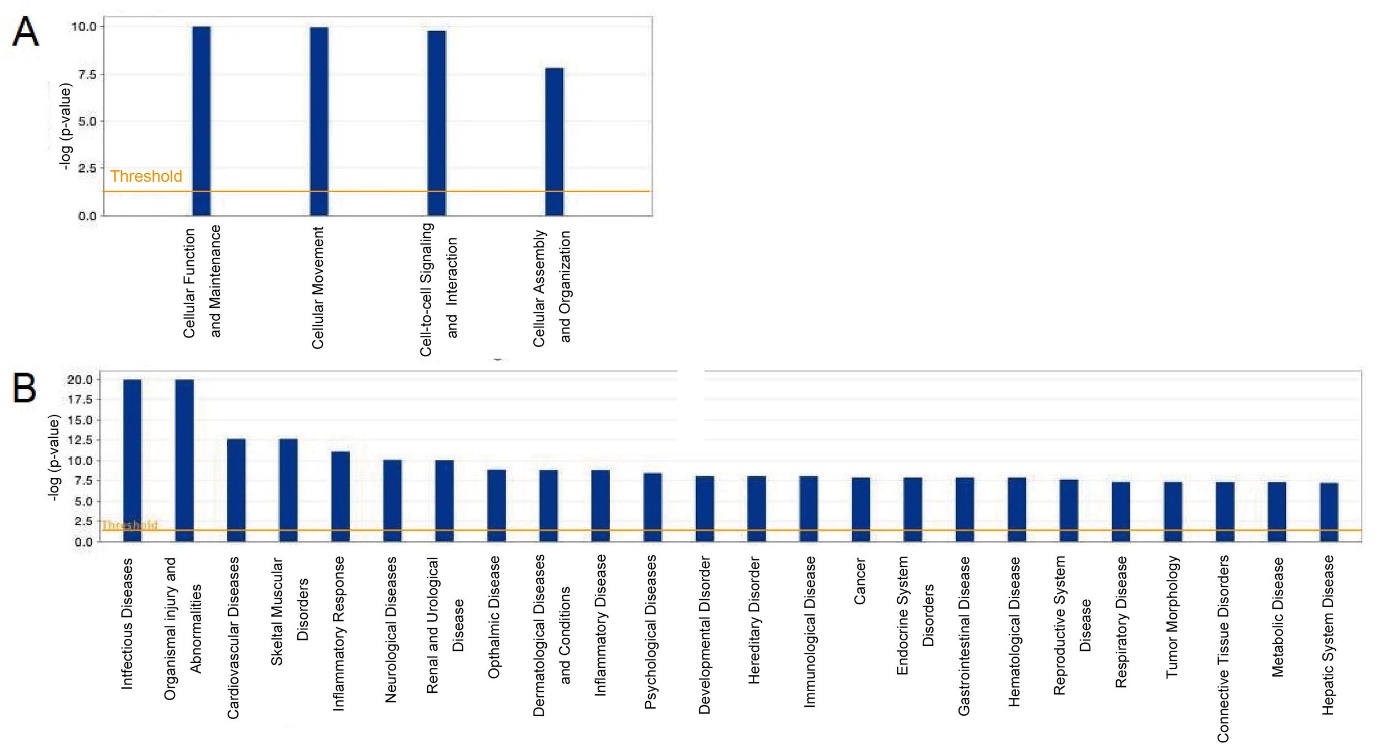


**Supplemental figure 2. UIP-specific network**

Bayesian network depicted UIP-specific networks, which are mainly consisted of the same modules as those in the IPF (UIP + pro-UIP) network, which included modules related to “TGF-β”, “fibrosis”, “complement”, “inflammation and myofibroblast proliferation”, “serological markers and clinical findings” and “tubulin”. Square nodes represent differential expressed proteins between the IPF group and control group. The size of nodes represents the number of edges leaving from the node, namely the number of outgoing orders. Black nodes represent protein, orange nodes represent blood tests,　green nodes represent EHR. Nodes painted red inside represent proteins upregulated in the IPF group compared to the control group, blue inside represents downregulated.


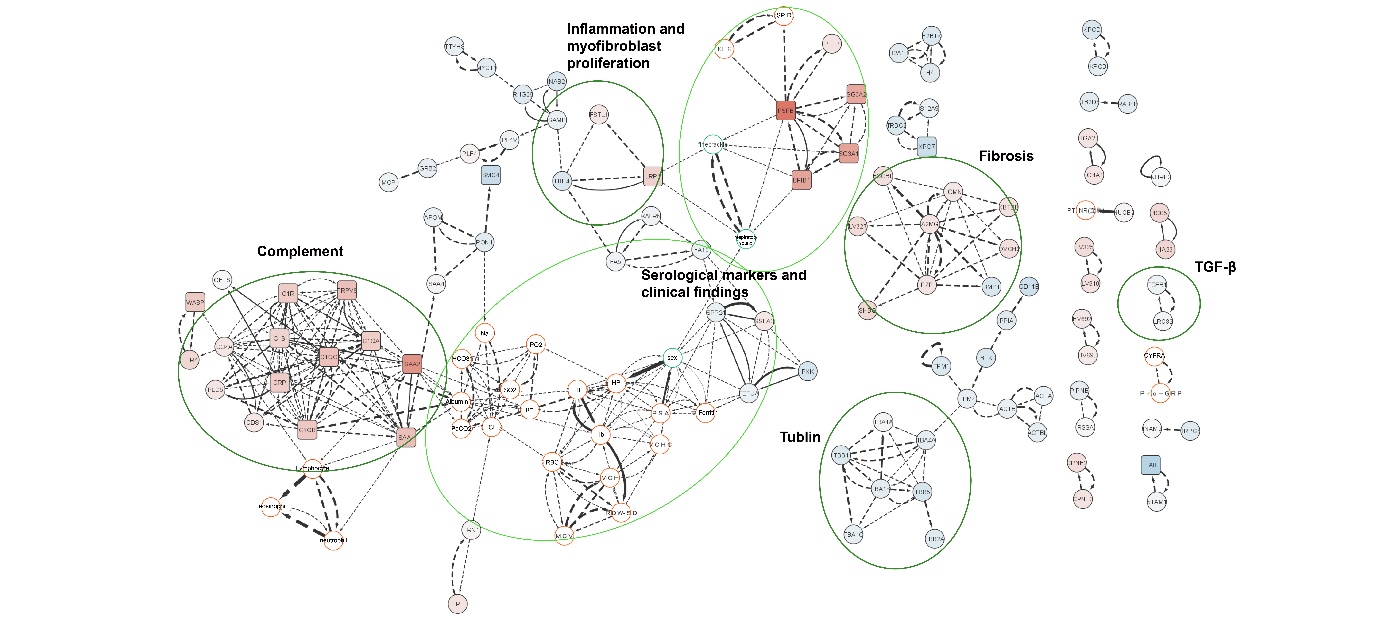


**Supplemental figure 3. IPF network mapping the difference in edges by sex and age.**

IPF network mapping the difference in edges by sex was shown in **(A)**. Solid lines are edges selected under the condition of the top 1% of ΔECv, dotted lines are edges connected to selected nodes and edges at distance=1. The width of the edge reflects the size of ΔECv. Square nodes represent differential expressed proteins between the IPF group and control group. The size of nodes represents the number of edges leaving from the node, namely the number of outgoing orders. Black nodes represent protein, orange nodes represent blood tests, green nodes represent EHR. Nodes painted red inside represent proteins upregulated in the IPF group compared to the control group, blue inside represents downregulated. Blue represents edges characteristic of male IPF patients, and purple represents edges that changed characteristically in both male and female IPF patients (corrected p-value < 0.05). Also, asterisks in the upper right corner of protein nodes indicate proteins with sex-dependent differences. Red * indicates differential expressed protein of the female IPF vs. female control groups, blue * indicates differential expressed protein of the male IPF vs. male control groups, and purple * indicates proteins with differential expression in both sexes (FDR < 0.05, |logFC| > 1.0). IPF network mapping the difference in edges by age was shown in **(B)**. Blue represents edges characteristic of young IPF patients, and purple represents edges that changed characteristically in both young and elderly IPF patients (corrected p-value < 0.05). Also, asterisks in the upper right corner of protein nodes indicate proteins with age-dependent differences. Red * indicates differential expressed protein of the elderly IPF vs. elderly control groups, blue * indicates differential expressed protein of the young IPF vs. young control groups, and purple * indicates proteins with differential expression in both age-groups (FDR < 0.05, |logFC| > 1.0).

**Supplemental Figure 3.**


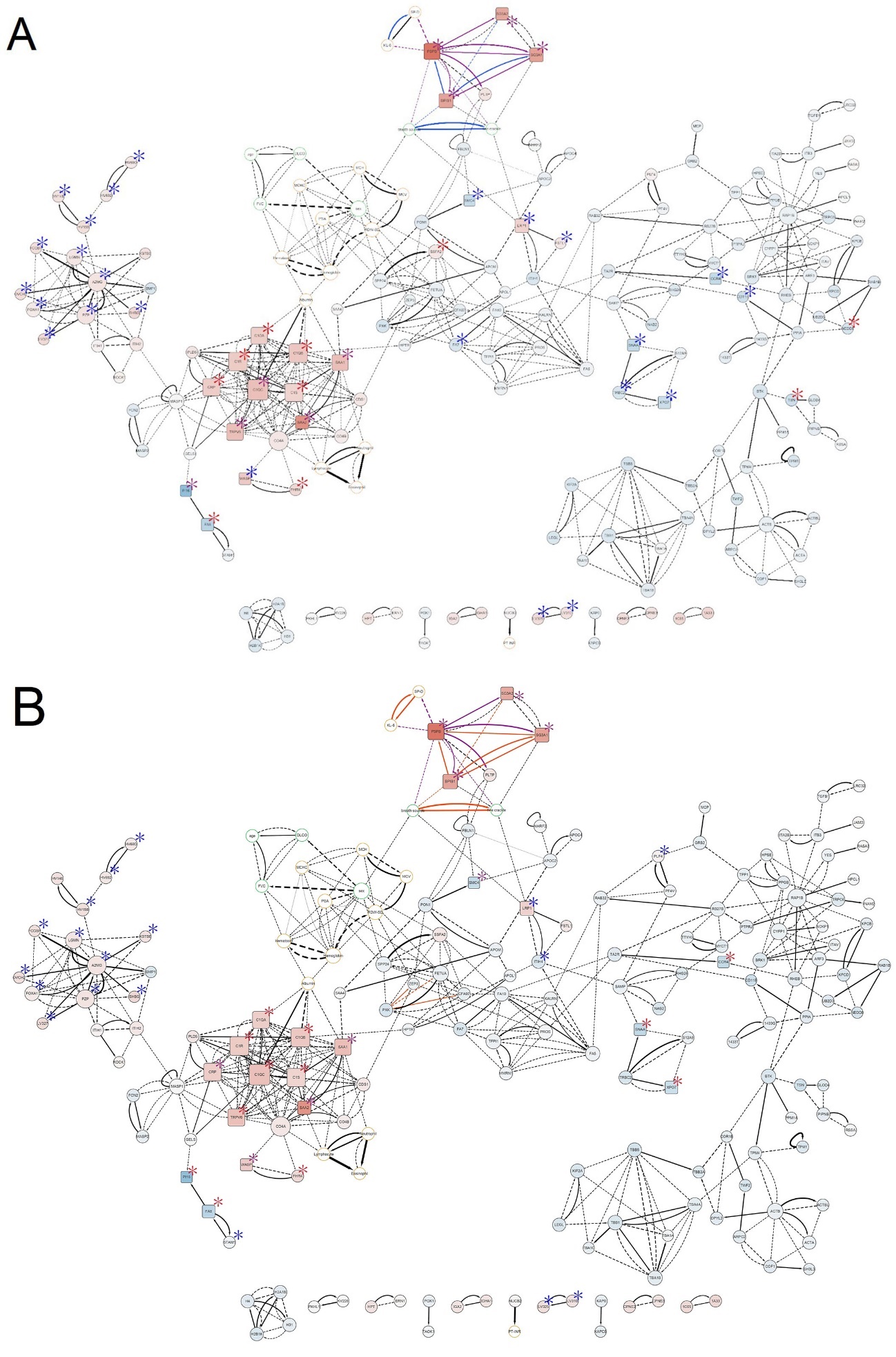


**Supplemental table 1. Basic statistics of the foundation network.**

| Edge type | Edge number | Proportion (%) | Child category | Edge number | Proportion (%) |
| --- | --- | --- | --- | --- | --- |
| Proteome → Proteome | 13162 | 88.57 |  |  |  |
| Blood test → Proteome | 15 | 0.10 | Proteome | 13178 | 88.68 |
| Template → Proteome | 1 | 0.01 |  |  |  |
| Blood test → Blood test | 853 | 5.74 |  |  |  |
| Proteome → Blood test | 362 | 2.44 | Blood test | 1300 | 8.75 |
| Template → Blood test | 85 | 0.57 |  |  |  |
| Proteome → Template | 94 | 0.63 |  |  |  |
| Blood test → Template | 143 | 0.96 | Template | 383 | 2.58 |
| Template → Template | 146 | 0.98 |  |  |  |
| Total | 14861 | 100.00 |  | 14861 | 100 |

The total number and proportion of each edge type are displayed. Edge types were defined by different combinations of parent-to-child categories. The template indicates data from the medical record template.
